# Cost of Investigating Neurological Disease: Experience of a Tertiary Care Center in Karachi, Pakistan

**DOI:** 10.7759/cureus.9291

**Published:** 2020-07-20

**Authors:** Sidra J Faruqi, Naila N Shahbaz, Qamar Nisa, Sumera R Umer, Syed G Ali, Muhammad Y Aziz

**Affiliations:** 1 Neurology, Hamdard University Hospital, Karachi, PAK; 2 Neurology, Dow University of Health Sciences, Karachi, PAK; 3 Medicine, Hamdard University Hospital, Karachi, PAK

**Keywords:** neurological disorders, economic burden, cost of investigations

## Abstract

Introduction: Neurological disorders, structural or functional, are prevalent all over the world and are accompanied by physical and social morbidity. In this study, we aimed to calculate the cost of investigating neurological disorders and compare the costs incurred in a government hospital with that in a private hospital.

Materials and Methods: This study was conducted at the Dr. Ruth KM Pfau Civil Hospital, Karachi, Pakistan. One hundred patients were enrolled in the study; 10 each investigated for epilepsy, cerebrovascular accidents (CVAs), headache, neuropathy, myopathy, cranial nerve palsies, movement disorders, demyelinating diseases, central nervous system (CNS) infections, and dementia. Receipts and records in the patients’ medical history were used for the calculation of the cost of procedures, which was then compared with the costs of these investigations in a private hospital. A bottom-up costing approach was taken with individual costs being estimated and then being grouped to calculate the overall economic burden of the disorders. Data were analyzed using IBM SPSS version 23.0. One-way analysis of variance (ANOVA) was done to compare the mean cost (taken by the patient, covered by the government, total cost in the government hospital, and total cost in the private hospital) across diseases in government and private hospitals separately. Pearson correlation and scatter plot were also done to study the cost in private and public hospitals. p-values less than 0.05 were considered statistically significant. The margin of error in the study was 5%.

Results: The mean age was 38.2 ± 20.5 years. Some 51% data were received from female samples. The mean income of samples was 13863.6 ± 9715.9 Pakistani Rupees (PKR) or 78.38 ± 58.29 United States Dollars (USD). The mean cost covered by government hospitals was 8866.0 ± 5071.0 PKR (53.19 ± 30.42 USD) per patient, whereas in government hospitals patients were charged on average 2662.9 ± 3774.7 PKR (16 ± 22.65 USD), while in private hospitals patients paid on average 29041.3 ± 12992.6 PKR (174.21 ± 78 USD).

Conclusion: The costs of investigations in private hospitals were approximately three times the costs in government hospitals. The maximum cost was generated by patients being investigated for demyelinating disorders. Investigations conducted in government-run hospitals are more cost effective and these institutions should receive increased funding to cater to the maximum number of patients.

## Introduction

Out of all the chronic diseases that burden the world, neurological or mental illnesses are the most prevalent [1]. The term ‘disorders of the brain’ is used to describe mental and neurological illnesses. Together, these make up approximately 13% of all diseases experienced by patients worldwide. This percentage is greater than the percentage of people suffering from cardiac diseases or malignancies [2]. The prevalence of neurological disorders ranges from 4% to 5% in lower income countries (such as Pakistan) as compared to 10% to 11% in high income countries [3]. Measurements of the all cause morbidity burden, calculated by disability-adjusted life years (DALYs), of neurological disorders in the European Union showed that disorders of the brain were the largest cause of morbidity and pose the greatest economic challenge for European healthcare [4]. Furthermore, the Global Burden of Disease studies that cover all disease groups and injury categories, demonstrate that, in the future, increasingly higher proportions of the global burden of disease will be attributed to brain disorders [5].

Due to inadequate resources available for health research, it is difficult to decide which disease should be given priority. Studies that investigate the cost of illnesses provide useful estimates of the financial and economic burden of specific diseases [6]. Therefore, by performing the cost of illness studies for several disorders, the financial and economic burden of these diseases may be calculated. This will, in turn, help researchers allocate funds for the diseases that are most prevalent and that cause the highest morbidity, social, and financial burden [6]. A 2006 United Kingdom (UK) Governmental review concluded that the impact of diseases on the population and economy should be studied in order to determine health research priorities [7].

Overall prevalence of epilepsy in Pakistan is estimated to be 9.99 per 1000 population [8]. The prevalence of stroke and/or transient ischemic attack (TIA) is 21.8% with 66.4% of the patients being female [9]. The estimated annual incidence of stroke in Pakistan is 250/100 000, translating to 350 000 new cases every year [10]. Another study, conducted in Faisalabad, Pakistan, concluded that 19.6% of the study population has depression, 16.6% epilepsy, and 15.2% suffer from migraines [11].

Pakistan had a gross domestic product (GDP) per capita of $1,340 in 2019, which ranks 147th in the world [12]. With nearly a quarter of the population living below the poverty line (Economic Survey 2018), it is difficult for patients to be able to spend funds on diagnosis and treatment of medical disorders.

The rationale of the study is to determine the cost of investigation of neurological disorders in a government-run tertiary care center in Karachi, Pakistan. It will ascertain the burden on the patients, the government-funded hospitals, and compare this cost to that of a reputed private institution. This will establish the local perspective as there is paucity of local data. The results of this study will be projected to various healthcare institutions and the health ministry so that appropriate facilities and funding for the management of neurological disorders may be allocated to government sector institutions.

The objective of this study was to calculate the cost of investigation of neurological disorders and estimate the burden on the patients as well as the government-funded hospitals and to compare this cost with the costs incurred in a private institution for the investigation of the same illness.

## Materials and methods

Study design: Retrospective study.

Study setting: Study was conducted at the Department of Neurology, Dr. Ruth KM Pfau Civil Hospital, Karachi.

Duration of study: Six months from 01-07-2018 to 31-12-2018. 

Sample size: Data from 100 patients were analyzed, 10 patients each from the major neurological disorders presenting to the department. The following neurological disorders were included in the study: epilepsy, cerebrovascular accidents (CVAs), neuropathy, myopathy, movement disorders, dementia, headache, demyelinating/inflammatory disorders, central nervous system (CNS) infections, cranial nerve palsies.

Sampling technique: Nonprobability consecutive sampling.

Sample selection:

*Inclusion Criteria*:

Either gender.

Presenting to the Dr. Ruth KM Pfau Civil Hospital Karachi, Pakistan for the first time without any prior investigations.

*Exclusion Criteria*:

Nonconsenting.

Patients previously investigated for the same disorder.

Patients availing funding from some outside source.

Cost analysis: Receipts and records in the patients’ medical history were used for the calculation of the cost of procedures. For the investigations conducted within the hospital, the prices were taken from the official list issued by the hospital. For the purpose of comparison with a private hospital, the official tariff list of a renowned private hospital was obtained. While the private hospital released their tariff list to us, they did not allow us to mention their name in the study. The cost was initially calculated in Pakistani Rupees (PKR) and was then converted to United States Dollars (USD). For the purpose of conversion, daily weighted average rate issued by the State Bank of Pakistan was used [13].

Economic evaluation: A bottom-up costing approach was taken with individual costs being estimated and then being grouped to calculate the overall economic burden of the disorders.

Statistical analysis: Data were stored and analyzed using IBM SPSS version 23.0. Count with percentages reported for baseline characteristics like age group, gender, and type of diseases. Mean with standard deviation given for quantitative variables like age in years, income, cost covered by government hospitals and private hospitals. One-way analysis of variance (ANOVA) was done to compare the mean cost (taken by the patient, covered by the government, total cost in the government, and total cost in the private hospital) across diseases in government and private hospitals separately. Pearson correlation analysis was done to see the relationship of treatment cost on diseases between government and private hospitals; the scatter plot was also done to show the trend of cost. There was 89% positive correlation between government and private sector cost. R-square showed 79.3% variation in private hospital cost was explained by the help of government sector cost. The p-values less than 0.05 were considered statistically significant. The margin of error in the study was 5%.

## Results

In this study there were 100 samples, 25% having age between 5 and 20 years. The mean age was 38.2 years. Some 51% data were received from female samples (Table 1).

**Table 1:** Baseline characteristics of studied samples (n=100).

Characteristics		n	%
Age group	5-20 years	25	25.0
	21-30 years	22	22.0
	31-45 years	18	18.0
	46-60 years	19	19.0
	>60 years	16	16.0
Gender	Female	51	51.0
	Male	49	49.0

Mean income of samples was 13863.6 ± 9715.9 Pakistani Rupee (PKR) or 78.38 ± 58.29 United States Dollars (USD). The mean cost in government hospitals was 8866.0 ± 5071.0 PKR (53.19 ± 30.42 USD) per patient, whereas patients were charged on average 2662.9 ± 3774.7 PKR (16 ± 22.65 USD), while in private hospitals patients paid on average 29041.3 ± 12992.6 PKR (174.24 ± 78 USD). The costs of investigations in private hospitals were three times the costs in government hospitals (Table 2). 

**Table 2:** Cost of investigations in public and private hospitals. PKR: Pakistani rupees; USD: United States dollars; SD: standard deviation

Cost			
Cost in government hospitals			
PKR	Mean ± SD	8866.0	±5071.0
USD	Mean ± SD	53.19	30.42
Taken by patients			
PKR	Mean ± SD	2662.9	±3774.7
USD	Mean ± SD	16	22.65
Covered by the Government			
PKR	Mean ± SD	7551.1	±3934.7
USD	Mean ± SD	45.30	23.61
Cost in private hospitals			
PKR	Mean ± SD	29041.3	±12992.6
USD	Mean ± SD	174.24	78

Results of one-way ANOVA showed mean and standard deviation of cost for each studied disease by the patient, in the government hospital, and in the private hospital. The mean cost for all diseases was statistically significant across type of diseases in government hospitals and private hospitals. The maximum cost incurred was for demyelinating disorders and the minimum for headache (Table 3). 

**Table 3:** Mean comparison of cost paid in government and private hospitals for studied diseases (n=100). SD: standard deviation; PKR: Pakistani rupees; USD: United States dollars; CVA: cerebrovascular accident; CNS: central nervous system *p < 0.05 was considered significant using one-way analysis of variance (ANOVA)

Diseases	Taken by patients		Covered by the Government		Total cost in government hospitals		Total cost in private hospitals	
	Mean ± SD		Mean ± SD		Mean ± SD		Mean ± SD	
	PKR	USD	PKR	USD	PKR	USD	PKR	USD
Epilepsy	611 ± 501	3.6 ± 3	4399 ± 2832	26.3 ± 17	4069 ± 2908	24.3 ± 17.4	16213 ± 9120	99 ± 54.5
Neuropathy	1050 ± 550	6.2 ± 3.29	4929 ± 1221	29.5 ± 7.3	5979 ± 1084	35.8 ± 6.5	27574 ± 4991	165 ± 30
Dementia	2337 ± 2267	14 ± 13.5	9799 ± 4502	58.6 ± 27	11074 ± 3154	66.3 ± 18.9	34225 ± 10898	204.8 ± 65.2
Myopathy	2571 ± 2768	15.4 ± 16.5	9507 ± 2730	56.9 ± 16.3	11564 ± 2509	69.2 ± 15	36912 ± 4408	221 ± 26.3
Cranial nerve palsy	7800 ± 1697	46.7 ± 10.1	4030 ± 4388	24.3 ± 26.2	7150 ± 5041	42.8 ± 30.1	20515 ± 13392	122.8 ± 80.1
CVA	6153 ± 5548	36.8 ± 33.2	9520 ± 2491	57 ± 15	11366 ± 2594	68 ± 15.5	35725 ± 4708	213.8 ± 28.2
Headache	2750 ± 1767	16.5 ± 10.5	2142 ± 1690	12.8 ± 10.1	2701 ± 1936	16.1 ± 11.5	10522 ± 6857	63 ± 41
Movement disorders	1070 ± 1286	6.4 ± 7.7	7351 ± 1763	44 ± 10.5	7044 ± 2302	42.1 ± 13.8	22048 ± 5384	132 ± 32.2
CNS infections	1100 ± 1090	6.6 ± 6.5	8720 ± 1675	52.1 ± 10	9270 ± 2048	55.5 ± 12.2	29839 ± 4973	178.6 ± 29.8
Inflammatory/Demyelination	6400 ± 6872	38.3 ± 41.1	9999 ± 5374	59.8 ± 32.1	15119 ± 8492	90.5 ± 50.8	45166 ± 17708	270.3 ± 106
p-Value	<0.01*		<0.01*		<0.01*		<0.01*	

**Figure 1:**
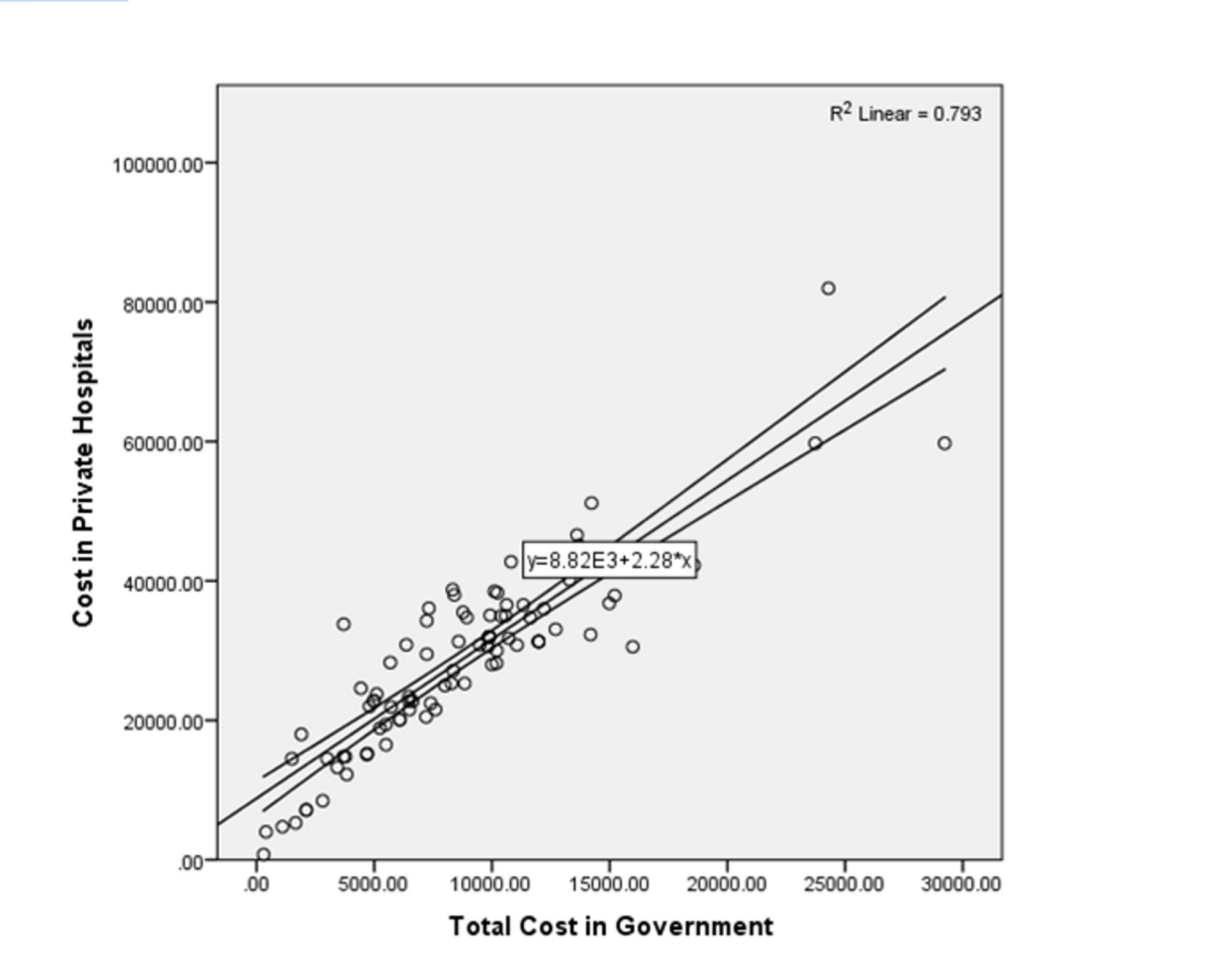
Scatter plot: correlation analysis of private and government sector cost. Straight lines show average trend for cost of private and government hospitals Curve lines given 95% confidence interval for correlation values Circles are the paired observation of diseases cost (government, private) R2 is coefficient of determination Equation is showing average increase in private hospital cost as per unit increase in government cost One unit increase in cost of government hospital will give on average 2.28 units increase in cost of private hospital

Scatter plot showed a positive correlation between the cost of government and private hospitals (Figure 1). As the cost of treatment increased in government hospitals it also increased in private hospitals. There was 79.3% variation explained in the cost of private hospitals by the help of the cost of government hospitals.

## Discussion

Health economics is defined as the application of economic theory, models, and empirical techniques to the analysis of decision making by individuals, healthcare providers, and governments with respect to health and health care [14]. While generally economics deals with the simple relationship between demand and production, health economics is complicated by the presence of a third-party agent, the physician, who decides what tests to order, what drugs to prescribe or what procedures to perform i.e. essentially making all the purchasing decisions for the consumer while not being affected by the cost of the product or the service required.

Costs of illness studies, like our study, are an integral component of health economics. These studies help calculate the cost of specific disorders on the economy thereby allowing important decisions to be made regarding resource allocation for these disorders. In addition to calculating the amount of money spent on a particular disorder, they also identify the different components contributing to this cost and highlight areas of potential research. 

There are three factors that contribute to the economic burden or cost of illness. Direct costs have the greatest contribution to the overall economic burden. These include the cost of hospitalization, investigations, and treatment given. Indirect costs measure the economic burden due to loss of productivity of an individual because of morbidity associated with the disease or early mortality. The final factor is termed tangible costs which represent the suffering, pain, and poor quality of life occurring as a result of disease [15-16].

In our study, we calculated the cost of investigating several common neurological disorders. According to our results, the diseases that required the greatest amount of money to investigate were demyelinating disorders, stroke, dementia, and myopathy. Cost of illness studies conducted by Andlin-Sobocki et al. (2005) and Pugliatti et al. (2008) showed similar results [17-18].

Our study estimated that the mean monthly household income of our patients was 13863 PKR (78.38 USD). The overall cost of investigation of a neurological disorder in a government hospital was 8866 PKR (53.19 USD) of which the patient only contributed approximately 30% whereas the remaining cost was paid by the government. So, in a government setting, the patient only required 19% of a single monthly income for the investigation of their disorder. However, if these same patients were investigated in a private hospital, they would be charged a total of 29041 PKR (174.24 USD) for diagnostic purposes, all of which they would have to pay themselves without any government contribution. This means that the patient’s entire household income for two months would just be spent in investigating their disease if they were to visit a private hospital for this purpose. Spending two months income merely on investigations is inconceivable for these patients.

Our study also concluded that the cost of investigations in a private hospital is nearly three times that of the cost in a government hospital. Also, in a government hospital, 70% of the cost is borne by the hospital making it easier for the patients to seek help for workup of their illnesses. Therefore, government hospitals require increased funding so as to facilitate the maximum number of patients who, if not for state-run institutions, would not have access to quality healthcare.

Various studies, conducted on stroke patients, have concluded that imaging and other laboratory investigations account for 11%-20.5% of the total economic burden of a disease [19-21]. Another study, conducted on a patient suffering from Parkinson’s disease, estimated that drugs and in-patient care accounted for 91% of the total cost of investigation and treatment of the disease [22]. In our study, we have focused solely on the cost of investigations. If we take into account the costs of drugs, rehabilitation, ward and doctors' fees, the estimated burden on both the patients and the government is much higher which leads us to reiterate the fact that state-run institutions should receive robust funding to adequately cater to the population, a quarter of whom live below the poverty line [12].

Our study has several limitations. First, we only calculated the cost of investigating the disease. The cost of in-patient care, medicines, rehabilitation (both in-patient and out-patient) figure significantly in the economic burden of any illness. Second, our results reflect the experience of a single hospital catering to a specific socio-economic group. Furthering this study to include more hospitals will increase the accuracy of our results. Finally, we only calculated the individual costs to the patient and the hospital. Projecting these figures in a true bottom-up fashion onto the prevalence of diseases will show us the actual economic burden of these disorders.

## Conclusions

The maximum cost was generated by patients being investigated for demyelinating disorders closely followed by CVAs, dementia, and myopathy. The minimum costs were incurred during investigation of patients with headache. Investigations conducted in government hospitals cost only one-third of the total amount spent in private hospitals. In government hospitals, patients were required to pay only a small fraction of the total charges and the rest was contributed by the hospital. Investigations conducted in government-run hospitals are more cost effective and these institutions require increased funding to adequately cater to the maximum number of patients.
